# Medication non-adherence: reflecting on two decades since WHO adherence report and setting goals for the next twenty years

**DOI:** 10.3389/fphar.2024.1444012

**Published:** 2024-12-23

**Authors:** Przemyslaw Kardas, Bryan Bennett, Bijan Borah, Michel Burnier, Christopher Daly, Mickael Hiligsmann, Enrica Menditto, Andrew M. Peterson, Julia F. Slejko, Krisztina Tóth, Elizabeth Unni, Tamás Ágh

**Affiliations:** ^1^ Medication Adherence Research Center, Department of Family Medicine, Medical University of Lodz, Lodz, Poland; ^2^ Patient Centred Outcomes, Jazz Pharmaceuticals, Oxford, United Kingdom; ^3^ Division of Healthcare Delivery Research, Mayo Clinic College of Medicine and Science and the Kern Center for the Science of Healthcare Delivery, Mayo Clinic, Rochester, MN, United States; ^4^ Faculty of Biology and Medicine, Switzerland and Hypertension Research Foundation, University of Lausanne, Lausanne, Switzerland; ^5^ Department of Pharmacy Practice, School of Pharmacy and Pharmaceutical Sciences, University at Buffalo, Buffalo, NY, United States; ^6^ Department of Health Services Research, CAPHRI Care and Public Health Research Institute, Maastricht University, Maastricht, Netherlands; ^7^ CIRFF, Center of Pharmacoeconomics and Drug Utilization Research, Department of Pharmacy University of Naples Federico II, Naples, Italy; ^8^ Philadelphia College of Pharmacy, Saint Joseph’s University, Philadelphia, PA, United States; ^9^ Department of Practice, Sciences and Health Outcomes Research, University of Maryland School of Pharmacy Baltimore, Baltimore, MD, United States; ^10^ Bridge of Health Alliance against Breast Cancer Association, Budapest, Hungary; ^11^ Syreon Research Institute, Budapest, Hungary; ^12^ Department of Social, Behavioral, and Administrative Sciences, Touro University, New York City, NY, United States; ^13^ Medication Adherence Research Group, Center for Health Technology Assessment and Pharmacoeconomic Research, University of Pécs, Pécs, Hungary

**Keywords:** medication adherence, drug therapy, innovation, digital health technologies, healthcare costs, patient education, patient outcomes, polypharmacy

## Abstract

**Background:**

Non-adherence to medication remains a persistent and significant challenge, with profound implications for patient outcomes and the long-term sustainability of healthcare systems. Two decades ago, the World Health Organization (WHO) dedicated its seminal report to adherence to long-term therapies, catalysing notable changes that advanced both research and practice in medication adherence. The aim of this paper was to identify the most important progress made over the last 2 decades in medication adherence management and to initiate a discussion on future objectives, suggesting priority targets for the next 20 years.

**Methods:**

This research used the WHO adherence model as a theoretical framework, categorizing adherence factors into five dimensions: health system, therapy, condition, patient-related, and socioeconomic. Ten international experts, five from Europe and five from the United States, were assigned to these dimensions and participated in structured online discussions. Initially, based on their desk reviews, experts identified significant achievements and future targets. They then ranked these items and provided feedback through several rounds, ensuring anonymity to minimize bias, ultimately reaching a consensus. This iterative process allowed for the creation of top-ten lists of past achievements and future targets for medication adherence management over the next 20 years.

**Results:**

Analysis of the top-ranked achievements affirms that notable progress has been made in medication adherence research and practice over the past 20 years, with increased awareness and a surge in dedicated scientific publications. Despite these advancements, non-adherence remains a prevalent issue, underscoring the need for the ongoing implementation of innovative solutions identified in this work, such as novel digital health solutions. Interdisciplinary collaboration and a holistic understanding of patient behaviours and socio-economic factors are crucial.

**Conclusion:**

While refraining from imposing a rigid “adherence Decalogue,” we are confident that this overview of recent achievements and the curated selection of future targets may provide a useful foundation for further discussions aimed at advancing medication adherence management. Our results call for a paradigm shift, advocating the repositioning of medication adherence on national agendas and underscoring the necessity for an adherence-supportive ecosystem that extends beyond mere patient support.

## Introduction

Non-adherence to medication seems to be the problem as old as medicine itself. In the 5^th^ century B.C., in one of his treatises, Hippocrates, the “Father of Medicine,” made a note: Keep a watch also on the faults of the patients, which often make them lie about the taking of things prescribed. For through not taking disagreeable drinks, purgative or other, they sometimes die. What they have done never results in a confession, but the blame is thrown upon the physician (Hippocrates, 2023).

No matter how difficult the non-adherent behaviour may be to understand, it remains highly prevalent even in the 21st century. Unfortunately, the consequences of medication non-adherence at the population level are severe. It is very difficult to estimate the costs associated with this problem, however, the available data are alarming. Non-adherence has been reported to generate €80–125 billions of potentially avoidable direct costs (such as hospitalizations and medication wastage) and indirect costs (including work productivity losses) in the European Union (European Commission/Medi-Voice, 2011). In 2016, the cumulative expense of non-adherence to prescription drugs reached approximately $529 billion in the U.S. (Watanabe et al., 2018), with additional costs per patient ranging from $5,271 to $52,341 (Cutler et al., 2018). Additionally, it is estimated to be associated to nearly 200,000 deaths annually in the European Union (European Commission/Medi-Voice, 2011) and 125,000 deaths per year in the U.S. (Kim et al., 2018).

What is even more important at the individual patient level, medication non-adherence creates a barrier that obscures the benefits of evidence-based medicine. As Robert B. Haynes, a pioneer in this field of research, sadly noted, “The full benefits of medications cannot be realised at currently achievable levels of adherence.” (Haynes et al., 2002). If this situation persists, medication non-adherence will continue to pose one of the major obstacles to the advancement of medicine.

Two decades ago, the World Health Organization (WHO) released a seminal report on adherence to chronic treatments (Sabaté, 2003). Although it was not the first publication in the field, it played an unprecedented role. The report marked a significant leap forward in raising awareness about medication non-adherence beyond a limited circle of researchers. With this report, simplified yet memorable statistics entered the public domain: 50% of patients being non-adherent to long-term therapies. Consequently, the issue of non-adherence could no longer be overlooked, prompting a shift in public perception. It transformed non-adherence from being seen merely as a problem of personal loss faced by individual patients to population-based problem requiring a more comprehensive understanding of societal consequences. It also increased the awareness about the impact of medication non-adherence on healthcare systems, payers, and pharmaceutical companies, urging the widespread implementation of effective interventions to both prevent and solve this problem.

However, despite half a century of dedicated research and growing understanding among the stakeholders, medication adherence remains far from perfect. A recent review revealed a high level of non-adherence among multi-morbid patients, ranging from 44.1% to 76.5%, as reported by the studies included (Foley et al., 2021). Although an international research project on adherence funded by the European Commission over a decade ago provided comprehensive policy recommendations for promoting medication adherence in the EU (Ascertaining barriers for compliance), the progress has been slow. Despite the availability of numerous effective interventions capable of enhancing medication adherence, only few are applied in real-world settings and even fewer are reimbursed across Europe (Ágh et al., 2022). Unsurprisingly, a recent report by the Organisation for Economic Co-operation and Development (OECD) demonstrates that medication adherence is not at the top of national health agendas, and a majority of the European countries are neither monitoring adherence nor taking regular actions to improve it (Khan and Socha-Dietrich, 2018).

Does the slow progress in increasing medication adherence undermine the value of the WHO report and suggest that it was a futile initiative? Does this mean that no advancements have occurred in the field since its publication? In this paper, we aim to assess the progress made over the last 2 decades and initiate a discussion on future objectives, suggesting priority targets for the next 20 years.

## Methods

To provide a firm theoretical basis for this research, the framework of determinants of medication adherence proposed by the WHO was used. This framework groups the factors affecting adherence into five interacting dimensions: 1) health system; 2) therapy; 3) condition; 4) patient-related, and 5) socioeconomic factors (Sabaté, 2003).

Ten international adherence experts, representing various backgrounds (e.g., academia, industry, patient organizations), fields of activity (pharmacy, medicine, health services research), and geographical regions (Europe and the United States), were invited by two moderators (TA, PK) to participate in this study, and all of them agreed. Based on their expertise, each of them was assigned to one of the five dimensions of the WHO adherence model. The allocation was structured to include two experts, one from Europe and one from the U.S., who were asked to present the results of their desk reviews within a particular dimension. Remote discussions, facilitated by predesigned online questionnaires, were structured iteratively as described below to enable fair ranking and prioritization of the most appropriate items. To minimize potential bias, the experts worked independently and did not know the identities of the other participants by the fourth round.

In the first round, the experts were requested to describe the three most important achievements in medication adherence which took place within 20 years from publication of the WHO report, within the dimension of their particular expertise. They were also asked to define the three most important targets to be achieved within the same dimension in the next 20 years to come. In the second round, the two experts within each dimension were asked to rank all six achievement items provided for the past, and six targets for the future. In the third round, the experts were asked to comment on and approve the results of this ranking, being informed of a potential overlap between the items. In the fourth and the final round, moderators assisted experts in addressing any potential disputes and facilitated the process of reaching consensus on the entire set of items collected for both past achievements and future targets. As a result of this process, top-ten lists of past achievements and future targets were created, with two items for each of the five WHO model dimensions.

## Results

### Achievements over the last 20 years

The top-ranked achievement items provided by the experts for the last 2 decades indicate that a notable progress has been made in adherence research and practice during this period. Although the problem of non-adherence remains unsolved, there is an evident increase in general awareness, and research interest, reflected in a boost of dedicated scientific publications (Kardas et al., 2023). Consequently, viable solutions are being formulated, tested, and, albeit infrequently, implemented. Table 1 outlines these accomplishments, which are also briefly discussed below.

**TABLE 1:** Top-ranked achievements in medication adherence management over the last 20 years.

Social and economic dimension
• Health policies aimed at addressing economic barriers to medication adherence
• Implementation of Value-Based Healthcare and Outcomes-Based Payment Models linking financial remuneration directly to patient outcomes

Therapy dimension
• Simplifying medication regimens and enhancing the ease of therapy administration
• Guidance and frameworks to encourage monitoring, analysis, and reporting of adherence data in the development of new therapies

Patient dimension
• Integration of patient-reported outcome measures for medication adherence
• Growing interest in understanding patient-related reasons for medication non-adherence

Condition dimension
• Recognition of the disease-related issues contributing to decreased medication adherence over time
• Development of the whole-person care concept based on the collaboration between physicians, nurses and pharmacists, positively impacting medication adherence

Healthcare system dimension
• Shift towards placing the patient at the forefront of clinical decision-making for better adherence management
• Digital health technologies and mobile applications to improve medication adherence

### Social and economic dimension

#### Health policies addressing economic barriers to medication adherence

Several countries have implemented health policies to address economic barriers to medication adherence. Wide introduction of generic drugs, enhancements in insurance coverage, and subsidies for essential medications have collectively worked to alleviate economic disparities, fostering a healthcare landscape where a broader population can access necessary treatments (Francois et al., 2023). These policy changes play a pivotal role in addressing structural barriers to adherence by facilitating access to medications. Following the implementation of the model of essential medicines list, introduced by the WHO and updated every 2 years, many governments, such as those in India and China, have focused their policies on rational use of medicines and access to more medications for a wider population (Kar et al., 2010), (Guan et al., 2011).

### Value-based healthcare and outcomes-based payment models

The contemporary healthcare landscape has witnessed a paradigm shift from a traditional volume-based model to an increasingly prevalent value-based approach, wherein the emphasis is placed on enhancing patient outcomes and overall wellbeing (Porter and Lee, 2013). The advent of value-based healthcare models marks a departure from conventional reimbursement structures by linking financial remuneration directly to patient outcomes. This innovative approach creates compelling economic incentives for healthcare providers to invest in interventions that specifically target and enhance medication adherence (Agarwal et al., 2018). Recognizing the pivotal role of adherence in achieving positive health outcomes, this economic policy paradigm encourages the strategic implementation of adherence management strategies such as Value-Based Insurance Designs (VBID) within certain health plans. Consequently, several U.S.-based health plans award prizes to providers when they achieve 80% or more adherence in their patients (Parekh et al., 2019).

### Therapy dimension

#### Simplifying medication regimen and enhancing ease of administration

A significant progress has been made by the pharmaceutical industry in developing novel therapeutic administration in some disease areas. For example, the increased availability and use of insulin pumps and, more recently, digitally-enabled devices such as, e.g., auto-injectors have been observed (Berget et al., 2019). In other therapy areas, ease of administration has been addressed through the development of simplified treatment regimes. These include *inter alia* multi-compound pills, targeting either one disease (combination drugs) or several conditions (so-called polypills), to reduce pill burden (Castellano et al., 2022), once-daily therapies, to reduce the number of dosing occasions, used in disease areas such as anticoagulants, HIV, hypertension and diabetes, once-monthly therapies for osteoporosis, and long-action injectables to replace daily pills (e.g., combination of long-acting cabotegravir and rilpivirine allowing bimonthly injections for the treatment and prevention of HIV) (Bares and Scarsi, 2022).

#### Development of guidance and frameworks to prompt monitoring, analysing and reporting of adherence data in the development of new therapies

Since the 1980s, an estimated 20%–33% of all approved drugs have been dose-adjusted after market authorisation, and 60%–80% of those adjustments were dose reductions (Heerdink et al., 2002). Non-adherence in clinical trials is widespread and can lead to erroneous estimations of efficacy and safety, as well as emergence of drug resistance (Blaschke et al., 2012). The publication of guidance documents was aimed at addressing the problem (Mantila et al., 2022) (Eliasson et al., 2020). For example, the FDA acknowledged that good adherence increases the power of a study and has provided guidance on strategies to support and control adherence in clinical trials (US Food and Drug Administration, 2019). Similarly, the European Medicines Agency has provided guidance on using estimands framework for accounting for medication adherence in estimating treatment efficacy (European Medicines Agency, 2017). However, these guidance documents have not yet translated into appropriate measurement, analysis and reporting of adherence in clinical trials which risk the potential approval and reimbursement of, and ultimately adherence to, medicines with misguided efficacy and safety expectations.

### Patient dimension

#### Measuring medication adherence with patient-reported outcomes measures (PROMs)

Dedicated PROMs have been developed to enable researchers and clinicians to use tailored medication adherence measures. Recent systematic review evaluated the evidence of as many as 121 different PROMs used to assess this issue in many different indications (Kwan et al., 2020). Although some of them do not meet all evidentiary criteria, PROMs represent a practical and cost-effective solution. These tools are straightforward to implement and could be easily integrated into routine clinical practice by nursing staff, pharmacists, or other healthcare professionals. This practical approach serves as an effective means of screening for non-adherence.

#### Integration of patient-reported outcome measures for medication adherence

There is also a growing interest in understanding patient-related factors contributing to medication non-adherence (Kvarnström et al., 2021), (Kardas et al., 2013). Consequently, various self-reported scales have been developed to identify the causes of non-adherence. Historically, adherence was often gauged based on pharmacy claims databases. Currently, however, the focus extends beyond simply knowing the rate of non-adherence. There is an increased interest in understanding the reasons behind it as this knowledge is crucial for developing and implementing appropriate interventions (Zekic et al., 2021). In fact, PROMs are the only measures that can capture individual reasons, both drivers and barriers, for adherence or non-adherence. Notably, several pharmaceutical companies and payers are now delving into the reasons behind patient non-adherence to medications, with the intention to proactively respond to them.

### Condition dimension

#### Recognition of the disease-related issues contributing to decreased medication adherence

Over the last 20 years, the two major challenges concerned recognition of the disease-related issues contributing to lower drug adherence over time and increasing the awareness and knowledge on adherence among providers. Indeed, the ability of healthcare professionals to recognise poor adherence in several medical conditions was considered as relatively low and the same was true for their ability to intervene (Clyne et al., 2016). In the last 2 decades improvements have been made to recognise a lot of disease-specific parameters, such as duration of the disease, intensity of symptoms and multi-morbidities, and to integrate them into patient management. This has led to the conclusion that poor adherence is not limited to asymptomatic diseases such as hypertension or dyslipidaemia but is a global problem affecting all patients with any disease, even those with highly symptomatic pathologies. One important step forward is the increased recognition of the role of comorbidities at all ages, but particularly in older adults, in whom cognitive deficits, which often remain undiagnosed, may play an important interfering role.

#### The concept of whole person care, embedded in collaboration among physicians, nurses, and pharmacists

Presently, there is a general movement to implement the concept of team-based care in clinical practice to support several aspects of patient management including medication adherence and persistence (Hopkins and Sinsky, 2022). Although this management model is still moderately implemented in many countries because of local regulations, it has been shown to contribute substantially to the improvement of the control of some diseases in other countries (Matsumoto et al., 2024), (Stephen et al., 2022). This approach is particularly effective for the long-term management of patients with complex medical conditions and a high pill burden (Onor et al., 2024). A recent analysis of studies conducted in low- and middle-income countries reveals that team-based care, coupled with education, single pill combinations, and reminders, proves more effective in supporting adherence and persistence than any single intervention (Ogungbe et al., 2021).

### Healthcare system dimension

#### Shift towards placing the patient at the forefront of clinical decision-making

There has been a notable shift towards placing the patient at the forefront of clinical decision-making. Enhanced communication, trust-building and patient education are now recognized as pivotal components, with a growing emphasis on empowering patients to actively participate in their treatment management by shared decision-making (Fiorillo et al., 2020), (Deniz et al., 2021). Additionally, healthcare systems are increasingly recognising the significance of behavioural interventions and considering patients’ preferences as integral factors in supporting medication adherence. This evolving approach reflects a comprehensive strategy which focuses not only on treatment but also an active involvement of patients, with consideration given to their values and preferences. Interestingly, one indirect consequence of this shift is the replacement of the previously used term “compliance” with “adherence” due to the paternalistic relationship between doctors and patients that the former term was often perceived to imply (Vrijens et al., 2012).

#### Digital health technologies and mobile applications to improve medication adherence

Technological advancements including digital health technologies have driven progress in medication adherence management. Mobile applications, wearable devices, and smart pill dispensers have transformed this domain by offering reminders, tracking medication usage, and providing real-time feedback to both patients and healthcare providers (Peng et al., 2020). These innovations empower patients, enhance communication between patients and healthcare providers, and ultimately improve medication adherence rates, as well as health outcomes. Recently, there has been an increasing trend where these technologies also incorporate “gamification” strategies to enhance patient engagement (Ghorbani et al., 2021). These strategies have not only been used in the clinical trial setting to measure medication use, but have also been tested and implemented in real-world practice, owing to their effectiveness.

## Targets for the future

Despite all the accomplishments described above, non-adherence remains a highly prevalent problem. Therefore, it becomes obvious that better implementation of available guidance, as well as new ideas and innovative solutions are imperative in the years to come. The evolving landscape of healthcare, advancements in technology, and the dynamic nature of patient needs necessitate a continual re-evaluation and adaptation of strategies. As we move into the future, challenges such as emerging therapeutic modalities, evolving patient demographics, and further integration of digital health solutions underscore the need for a forward-looking approach.

Moreover, the complexities of modern healthcare systems demand interdisciplinary collaboration and a holistic understanding of patient behaviours, preferences, and socio-economic factors. This highlights the importance of fostering a culture of continuous innovation, where patients, researchers, clinicians, and healthcare stakeholders collectively contribute to the development of adaptive solutions. In fact, while the past 2 decades have seen remarkable strides in adherence research and practice, the journey toward enhancing patient outcomes through improved adherence is an ongoing and dynamic process. Embracing new ideas and solutions will be pivotal in navigating the complexities of healthcare delivery, ensuring that patients receive the best possible care tailored to their individual needs, and fostering a healthcare environment that is both responsive and resilient in the face of future challenges. Therefore, presented below are the results of the iterative selection of the ten most important targets for the next 20 years, also summarised in Table 2.

**TABLE 2:** Top-ranked targets for medication adherence management over the next 20 years.

Social and economic dimension
• Implementation of comprehensive measures of medication adherence, establishing a stronger connection between adherence metrics and health outcomes
• Thorough examination of the financial implications of medication adherence

Therapy dimension
• Advancing the role of digital technology in improving adherence
• Appropriate management of adherence in the context of polypharmacy and multimorbidity

Patient dimension
• Provision of patient education to enhance their understanding of their illness and medications
• Incorporation of medication adherence as a standard measure in routine clinical practice

Condition dimension
• Implementation of new technologies to support adherence and persistence, directly targeting pathogenic mechanisms contributing to disease development
• Improved identification of patients at high risk of non-adherence in clinical practice

Healthcare system dimension
• Using artificial intelligence in medication adherence management
• Increased access to adherence-enhancing interventions through the expansion of reimbursement and insurance coverage

### Social and economic dimension

#### Towards a comprehensive measure of medication adherence

The future landscape of medication adherence measurement necessitates an advanced approach that extends beyond the current standard measures, such as the Proportion of Days Covered (PDC), Medication Possession Ratio (MPR) or patient reports via dedicated PROMs tools (Rickles et al., 2003). While all these measures provide a quantifiable metric, there is a growing recognition that they fall short in capturing the complexity of medication adherence (Lam and Fresco, 2015). It is imperative to move beyond merely measuring the quantity of medication taken and delve into the qualitative impact on health. Future advancements in this field call for development and implementation of more robust measures - ones that not only reflect the “statistical” aspect of medication non-adherence but also incorporate behavioural, social, and patient-specific factors. Furthermore, the future of medication adherence research requires a stronger link between adherence metrics and health outcomes. By defining more comprehensive measures that integrate patient engagement, health literacy, and digital health technologies, coupled with a strong linkage to health outcomes, we can foster a patient-centred approach that goes beyond the limitations of current adherence metrics. This evolution holds great promise for the future as it may not only enhance adherence but also improve overall health outcomes for individuals managing chronic conditions.

#### Holistic Exploration of financial impact

The future of medication adherence necessitates a comprehensive examination of its financial implications, recognising the profound influence that cost-related factors can have on medication-taking behaviours among patients. Copayments and overall costs associated with prescribed medications can build substantial barriers to adherence, particularly in some healthcare systems. Current research underscores the negative impact of increased copayments on medication adherence and the benefit of no or low copayments, particularly among individuals with chronic conditions (Choudhry et al., 2014), (Schikowski et al., 2022). Therefore, understanding the intricate relationship between financial constraints and adherence is of paramount importance in developing strategies that reduce economic barriers for patients. Moreover, there is an imperative to determine the cost-effectiveness of interventions aimed at improving medication adherence. Evaluating the economic impact on both individual and societal levels is essential for crafting interventions that not only improve adherence but also generate substantial value for healthcare systems. By addressing the economic dimensions of adherence, we can advance strategies that promote affordability, enhance medication-taking behaviours, and contribute to the overall efficiency of healthcare delivery systems.

### Therapy dimension

#### Advancing the role of digital technology in adherence management and personalised medicine

An important development over the next 20 years will be to engage patients with digital technology to integrate continuous monitoring of treatment adherence, clinical outcomes, and side effects. This will allow for personalised reassessment of treatment recommendations, and thus an opportunity to minimise adverse effects through titration and treatment optimisation. For example, digital inhalers have an integrated electronic module for recording, storing and communicating with a mobile application and dashboard inhaler usage data, which means that they can give patients and their physicians feedback on the patient’s inhaler technique as well as their adherence (Kaplan et al., 2023). Additionally, more consideration should also be given to adherence to prescription digital therapeutics (PDTx), which are health software solutions intended to treat or alleviate a disease, disorder, condition, or injury by generating and delivering a medical intervention that has a demonstrable positive therapeutic impact on a patient’s health. Several of them have already been approved in both Europe and the U.S. (Wang et al., 2023). However, in the case of PDTx, non-adherence is likely to be an issue, just as it is with traditional medicines.

#### Appropriate management of adherence in the context of polypharmacy and multimorbidity.

The prevalence of multimorbidity and related polypharmacy are on the rise, mostly due to ageing of the global society. Unfortunately, polypharmacy paves the way to non-adherence (Franchi et al., 2021). Therefore, in the next 20 years more attention should be paid to the management of multiple health conditions rather than single diseases. In parallel, a consensus should be reached on the measurement and optimisation of adherence in patients taking multiple medications. Progress in the measurement and support for adherence in persons with multimorbidity could significantly improve outcomes for individual patients and reduce costs for healthcare systems. It may also address health inequalities, since people of lower socio-economic status, those representing minority ethnic groups and patients with severe mental illnesses are more likely to be affected by multiple health conditions (Álvarez-Gálvez et al., 2023).

### Patient dimension

#### Provision of patient education to enhance their understanding of their illness and medications

Patient having knowledge about their illness and medicines is one of the important patient-centred factors in medication adherence. At the same time, physicians and other healthcare providers claim that they do not have enough time to devote it to adherence support of their patients. Thus, pharmaceutical companies, health insurance and public health programs should be encouraged to become more innovative in providing patient education, enhancing overall health literacy and addressing misconceptions and concerns about illness and treatment. The current patient education methods such as brochures are not adequate. More resources should be spent to understand effective patient education strategies. To respond to adherence challenges successfully, it is crucial to harness advancements in behavioural sciences and health psychology. Exploring options like call centres, supported by pharmaceutical and health insurance companies, in medical, pharmacy, and nursing schools could be a proactive step to offer support to patients and enhance their understanding of medications. Notably, the World Health Organization (WHO) has recently released a dedicated guide on therapeutic patient education for policymakers, health professionals, and educational/training bodies (WHO).

#### Medication adherence should become one of the vital measures in routine clinical practice

To incorporate it seamlessly into intake procedures, nurses or physician assistants should systematically inquire about patients’ adherence to each prescribed medication in non-judgemental way, recording all the information, including reasons for non-adherence, in the Electronic Medical Record (EMR). This detailed documentation can serve as a valuable resource for physicians to engage in meaningful discussions with patients regarding their medication regimen. Recognising it as a vital measure will help keep track of the adherence behaviour with chronic medications (Magid and Ho). As far as patient-related factors are concerned, this meticulous documentation stands as the primary method for monitoring and providing timely interventions, also ensuring a comprehensive approach to healthcare management.

### Condition dimension

#### Implementation of new technologies to support adherence and persistence

Further development is now expected that could contribute to increasing adherence and persistence in several clinical conditions, acting directly on pathogenic mechanisms that contribute to the disease progress. This involves, for example, RNA-based therapies which are currently used in the treatment of dyslipidaemia, and are under development for hypertension and other diseases (Ren et al., 2020). It is hoped that these approaches will help to reduce practical barriers to adherence and persistence, allowing for drug administration once every 6 months or even once a year.

#### Improved detection of patients at high risk of non-adherence in clinical practice

Detection of patients at high risk of poor adherence and persistence is a major challenge in individuals with chronic health conditions. In recent years, new technologies have been developed to screen patients with chronic treatments for adherence. This includes, for example, the measurement of drug levels in blood or urine using LC-MS technologies, or the introduction of ‘digital pills’ equipped with ingestible microsensors (Browne et al., 2018). These approaches, which have some limitations, can be applied by research centres but are of limited use in clinical practice (Berra et al., 2016), (Peeters et al., 2024). Therefore, there is a clear need to develop new approaches that would enable physicians or healthcare professionals to detect patients at risk of non-adherence using simple but reliable methods. Notably, the same patient may exhibit different levels of adherence to various drugs, and at different time points throughout their journey (Schulz et al., 2016). Therefore, this approach should be applied to address each condition on a case-by-case basis.

### Healthcare system dimension

#### Using artificial intelligence in medication adherence management

Artificial Intelligence will play a pivotal role in medication adherence management, enabling healthcare systems to anticipate adherence barriers and intervene proactively. AI-powered chatbots and virtual assistants may deliver timely reminders, provide medication education, and offer supportive counselling, thus improving patient engagement and adherence outcomes (Babel et al., 2021). Moreover, real-time monitoring through wearable sensors and other devices will enable continuous assessment of medication adherence and prompt AI-enhanced interventions when deviations occur, with the aim of improving medication management and health outcomes. With the progress of data science and the integration of vast amounts of data from different sources, such as socioeconomic data, patient baseline clinical characteristics, patient-reported perceptual and practical barriers to adherence, and prescribing and dispensing data, it will be possible to predict quite accurately the medication adherence trajectory for an individual and create dynamic intervention plans to improve medication adherence. However, it is important to acknowledge that although application of ‘big data’ along with machine learning and artificial intelligence holds great promise for identifying patients for which adherence-improving interventions are helpful (Kardas et al., 2020), there also exists a potential risk that these technologies will be wrongly used to limit access to medications and healthcare. Furthermore, there needs to be an evidence-based consideration of which predictor variables should be included in these algorithms so as not to introduce bias and to support equitable decision making. Therefore, in the coming decades, ethical use of these technologies will be important.

#### Increased access to adherence-enhancing interventions

It is also expected that healthcare systems will significantly increase access to adherence-enhancing interventions by expanding reimbursement and insurance coverage. This improved access, ideally with reduced or no cost incurred by patients, will be pivotal in ensuring the use of these interventions and delivering comprehensive support to individuals (Kardas et al., 2022). Furthermore, telehealth and remote monitoring technologies will play a transformative role in broadening access to adherence-enhancing interventions beyond conventional healthcare settings. However, achieving equitable access to telehealth services and the necessary technology infrastructure will be paramount.

## Conclusion

Results of our study affirm that the WHO adherence report, published 2 decades ago, initiated a cascade of important changes. They have notably influenced both the research and practice of supporting medication adherence. However, despite these strides, we are still confronted with the persistent challenge of non-adherence. Considering the inherent complexities of human behaviour and the intricate network of other factors contributing to non-adherence, this problem is expected to remain in the years to come.

The multifaceted nature of the phenomenon highlights the fact that there is no universal answer or one-size-fits-all solution. To ensure further progress, a step-by-step strategy appears to be the most promising option. Within the spectrum of available approaches, careful selection becomes crucial, thus improving chances of success. While in this paper we have proposed a selection of targets for the future, we acknowledge the inherent limitations of this an approach, particularly due to the non-random selection of invited contributors. Notably, all experts came from high-income countries, which reflects the concentration of studies on this subject in Europe and the United States. However, this limits the diversity of perspectives, leaving low- and middle-income countries (LMICs) underrepresented. The accepted framework allowed for the presentation of only selected items, leaving no room for other issues that may be of significant importance in specific contexts, such as, e.g., drug shortages. For all these reasons, we do not intend to impose an ‘adherence Decalogue.’ Nonetheless, we believe that offering a carefully chosen set of top-ranked objectives identified by experts may provide a useful starting point for further discussions on the priorities of advances in medication adherence management.

In essence, these ten suggestions fuse together to call for a paradigm shift - a repositioning of adherence on national agendas. What is needed is not only patient support but also an overarching adherence-supportive ecosystem, which starts with accurate assessment of adherence in clinical trials, actively engaging patients and their organisations, and extends throughout every step of the patient’s therapeutic journey. In 2003, the WHO report quoted the statement by Haynes et al. (2002): “Increasing the effectiveness of adherence interventions may have a far greater impact on the health of the population than any improvement in specific medical treatments”. Undoubtedly, this phrase remains a guiding principle for those dedicated to addressing the challenges of medication adherence.

## Data Availability

The raw data supporting the conclusions of this article will be made available by the authors, without undue reservation. Requests to access the original datasets should be directed to przemyslaw.kardas@umed.lodz.pl.
